# Retracted: An Inverse Spectral Problem for the Sturm-Liouville Operator on a Three-Star Graph

**DOI:** 10.1155/2014/389481

**Published:** 2014-10-28

**Authors:** International Scholarly Research Notices

This paper [1] has been retracted as it is essentially identical in content with the published article “Determination of Sturm-Liouville operator on a three-star graph from four spectra,” by Dehghani Tazehkand and Akbarfam, published in Acta Universitatis Apulensis No. 32/2012, pp. 147–172.
